# One-Year Follow-Up of Subthalamic Nucleus Deep Brain Stimulation in SNCA Mutation Parkinsonism: A Case Report

**DOI:** 10.5334/tohm.819

**Published:** 2023-12-21

**Authors:** Eve Fouarge, Gaëtan Garraux, Bruno Kaschten, Anne-Laure Salado, Eric Parmentier

**Affiliations:** 1 Neurology department, University Hospital of Liège, Liège, Belgium; 2 Neurosurgery department, University Hospital of Liège, Liège, Belgium

**Keywords:** Monogenic Parkinson’s disease, SNCA mutation, deep brain stimulation, subthalamic nucleus

## Abstract

**Background:**

Deep brain stimulation (DBS) has shown some efficacy in monogenic Parkinson’s disease; however, data about its long-term benefit in SNCA mutations remain scarce.

**Case report:**

Subthalamic nucleus DBS was implanted in a 60-year-old female patient with Parkinson’s disease due to SNCA duplication. One year later, the patient walked unassisted and was independent for most activities of daily living, without requiring any anti-Parkinson’s medication.

**Discussion:**

To our knowledge, four cases of bilateral subthalamic DBS have been reported previously. This case report adds an additional body of evidence of improved one-year outcome after DBS surgery in a patient with SNCA mutation.

**Highlights:**

This is a case report of a patient with genetic parkinsonism due to SNCA duplication undergoing bilateral subthalamic nucleus (STN) deep brain stimulation (DBS) surgery. The outcome was favorable one year after implantation, with the patient coming off all anti-Parkinson’s medications. This further clarifies DBS outcome in monogenic Parkinson’s disease.

## Introduction

SNCA mutations cause genetic Parkinson’s disease (PD), that may be typical or present with atypical features such as early onset, hallucinations, dysautonomia, cognitive deficits and early motor complications [1,2]. Duplications often outlie the SNCA gene locus, and may contain over 100 genes [1,3]. Deep brain stimulation (DBS) is indicated in levodopa-responsive PD patients suffering from motor fluctuations and has shown some efficacy in monogenic PD. However, data about its long-term benefit in SNCA mutation is scarce, and should be strengthened, since SNCA-related Parkinson’s disease patients often exhibit non-motor symptoms [4,5].

## Case Description

We hereby report the case of a female patient who presented with rest tremor of the left arm and akinetic-rigid syndrome at the age of 53, four years before referral to our hospital, where the final diagnosis of genetic Parkinson’s disease due to SNCA duplication was made.

She had a developmental disability, mainly with learning difficulties, and she attended a special school, but this was not further investigated during her childhood. Her deceased mother may have had a tremor but was not investigated. Our patient initially consulted in another academic center, where a probable PD diagnosis was made and levodopa (LD)/benserazide 100/25 three times per day was initiated with a clear clinical benefit. However, after a few months, symptoms worsened and the dose of levodopa was increased to 200 mg three times per day, along with the addition of controlled-release (CR) LD/benserazide 100/25 and pramipexole extended-release 1.05 mg. The patient rapidly developed hallucinations, orthostatic hypotension and levodopa-induced dyskinesias, which prompted a decrease, then a pause, in PD medications. Levodopa was finally resumed to alleviate akinetic-rigid syndrome, in combination with tiapride to reduce dyskinesias.

**Figure 1:**
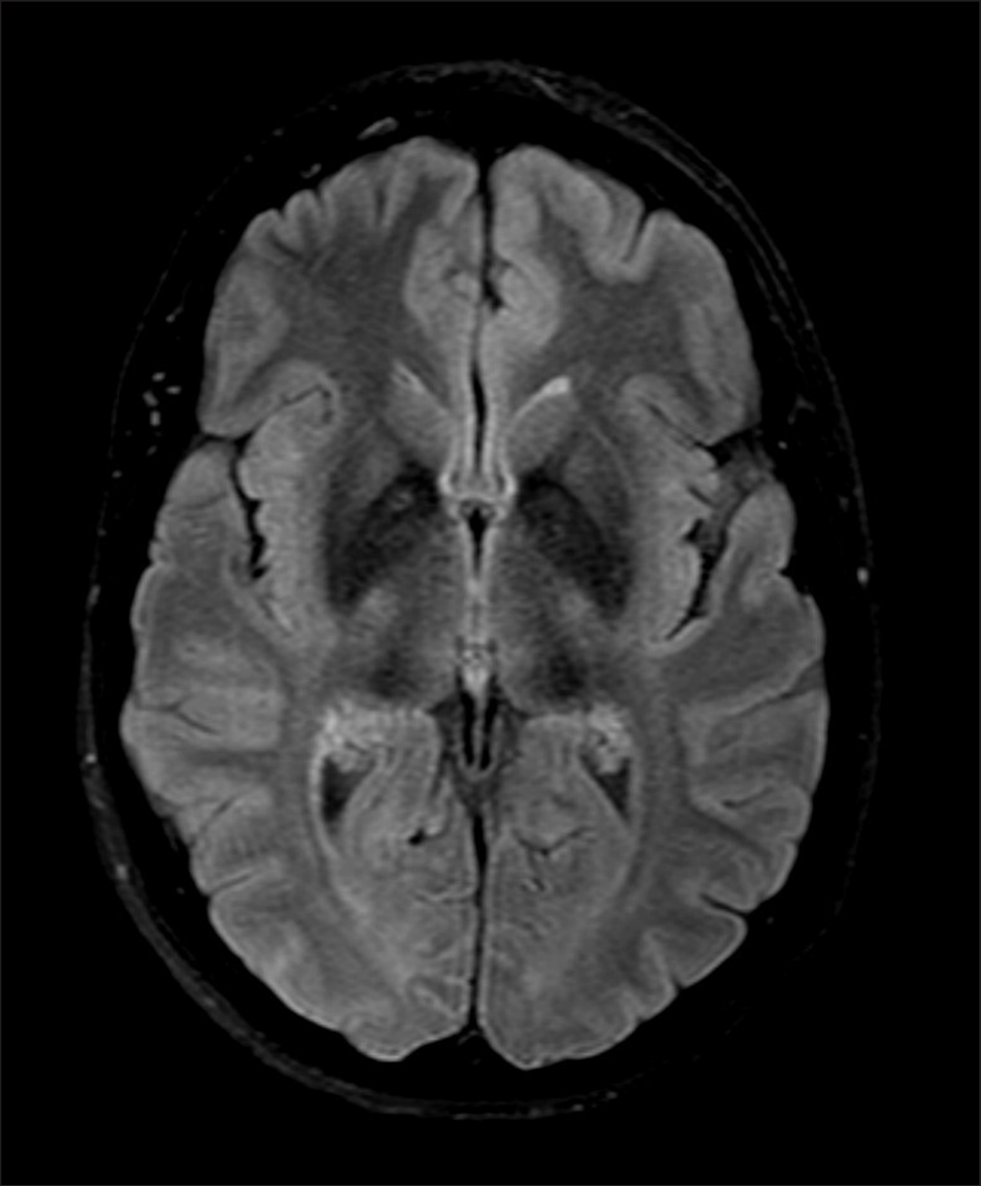
Brain MRI 2 years after symptom onset.

At that point, she complained of urinary incontinence and mild memory loss. This unusual evolution prompted additional exams; an ioflupane scintigraphy confirmed severe bilateral striatal dopaminergic denervation and a brain MRI showed a T2 hypointensity of the lenticular nuclei bilaterally (see Figure 1). Based on this, she was initially diagnosed with pantothenate kinase-associated neurodegeneration (PKAN), although the genetic testing was negative. Neurodegeneration with brain iron accumulation (NBIA) or multiple system atrophy (MSA) were suspected as alternate diagnoses. The patient was subsequently referred to our center.

At the time of referral, the patient had no treatment and could not walk without aid. She had severe rest tremor, predominant on the left side, severe akinesia and rigidity. Physical examination also showed enhanced deep tendon reflexes in all four limbs, left ankle clonus and a polykinetic right Achilles reflex. She had constipation, rapid eye movement sleep behavior disorder, hyposmia and persistent orthostatic hypotension. Levodopa was introduced again, in parallel with fludrocortisone, leading to mild improvement. Subsequent increases in levodopa daily dose resulted in a good motor response but also recurrence of disabling dyskinesias and hypotension, while decreases resulted in exacerbated akinesia, rigidity and tremor (see video 1). A follow-up MRI done 6 years after the first did not confirm any lesion of the basal ganglia and the lesions on the first imaging were considered a probable artifact. Neuropsychological testing indicated a below normal pre-morbid level of intellect as well as depressive symptoms. Episodic memory was preserved, and there was a slight reduction of verbal inhibition and verbal fluency. The patient complained of concentration difficulties but no memory loss and no decline. MIBG scintigraphy showed evidence of sympathetic cardiac denervation. Genetic testing showed a duplication of 11 Mb including the SNCA gene locus, which was suspected to be de novo (the patient’s parents were deceased).

**Video 1:** **Comparison of MDS-UPDRS III scores before and after surgery.** UPDRS III items 3.9 (arising from chair), 3.10 (gait) and 3.12 (postural stability) pre-surgery in ON and OFF state and one year after DBS surgery.

Considering her fair motor response to levodopa (see Table 1) and the absence of MRI contra-indications or marked cognitive decline, the patient underwent bilateral subthalamic nucleus (STN) deep brain stimulation (PERCEPT PC, Medtronic Inc) at the age of 60, following a procedure described previously [6]. The initial response was good with the following stimulation parameters: on the right, monopolar stimulation, contact 9-, intensity 1.1 mA, frequency 130 Hz, pulse width 60 microseconds; on the left, monopolar stimulation, contact 1-, intensity 1.3 mA, frequency 130 Hz, pulse width 60 microseconds. Contacts are labelled from 0 to 3 in a ventrodorsal direction on the left electrode, and from 8 to 11 in a ventrodorsal direction on the right electrode.

**Table 1:** MDS-UPDRS score and Hoehn & Yahr scale before (OFF and ON) and after DBS surgery (4 months and 1 year).


	PRE-DBS: OFF	PRE-DBS: ON	DBS ON (4 MONTHS)	DBS ON (1 YEAR)

**UPDRS-I**	**NA**	**23**	**/**	**10**

**UPDRS-II**	**45**	**21**	**/**	**16**

**UPDRS-III**	**72**	**38**	**17**	**19**

3.1 Speech	2	1	0	1

3.2 Facial expression	2	2	1	2

3.3 Rigidity				

*Neck*	*2*	*0*	*0*	*0*

*Upper limb (right/left)*	*3/2*	*0/0*	*1/0*	*1/0*

*Lower limb (right/left)*	*3/2*	*1/1*	*0/0*	*0/0*

3.4 Finger tapping (right/left)	3/2	1/ 1	1/1	1/1

3.5 Hand movements (right/left)	2/1	2/0	1/1	2/2

3.6 Pronation-supination movements of hand (right/left)	3/2	1/3	1/0	0/1

3.7 Toe tapping (right/left)	3/3	3/3	3/2	2/2

3.8 Leg agility (right/left)	1/2	1/2	1/1	1/0

3.9 Arising from chair	3	3	0	0

3.10 Gait	3	2	0	1

3.11 Freezing of gait	3	2	0	0

3.12 Postural stability	3	3	0	0

3.31 Posture	3	3	0	1

3.14 Global spontaneity of movement	3	2	1	1

3.15 Postural tremor of the hands (right/left)	2/1	0/1	0/0	0/0

3.16 Kinetic tremor of the hands (right/left)	0/0	0/0	0/0	0/0

3.17 Rest tremor amplitude				

*Lips & jaw*	*1*	*0*	*0*	*0*

*Upper limb (right/left)*	*2/2*	*0/0*	*1/0*	*0/0*

*Lower limb (right/left)*	*2/2*	*0/0*	*0/0*	*0/0*

3.18 Constancy of rest tremor	4	0	1	0

Dyskinesias during examination? Interfered with examination?		Yes.		

**UPDRS-IV**	**NA**	**14**	**0**	**0**

**Hoehn & Yahr**	**4**	**3**	**2**	**2**


Before surgery, UPDRS score was 38 on dopaminergic treatment (see Table 1) and Hoehn and Yahr scale was stage 3. The patient was treated with LD/benserazide four times per day (50/12.5–25/6.25–25/6.25–25/6.25 mg), CR LD/benserazide 100/25 mg at bedtime, safinamide 100 mg one time per day, amantadine 100 mg two times per day and fludrocortisone 400 micrograms one time per day. LD was completely interrupted following surgery as the patient still suffered from orthostatic hypotension despite being treated with fludrocortisone, as well as dyskinesia. There were no hallucinations or psychosis. Safinamide and amantadine were gradually interrupted in the following months and fludrocortisone was reduced to 20 micrograms one year after surgery.

Eight months after the surgery, the patient developed left brachiocrural hemiparesis (with a slight drop of the upper limb but no hollow-hand sign or weakness at the elbow, and a slight drop of the lower limb, without gait abnormalities), following SARS-CoV-2 infection a few weeks earlier. Spinal and brain MRIs could not explain the deficit. Interrupting stimulation did not impact the hemiparesis, but the patient recovered spontaneously in the following weeks.

One year after surgery, following repeated adjustments, stimulation parameters were the following: on the right, segmented stimulation in an anterior and medial direction on contacts 9- (0.5 mA), 10- (2.3 mA) and 11- (1 mA), frequency 150 Hz, pulse width 40 microseconds; on the left, double monopolar stimulation on contacts 2- (2.1 mA), 3- (0.6 mA), frequency 150 Hz, pulse width 40 microseconds).

The patient walked unassisted and was independent for most activities of daily living, without any anti-Parkinson’s medication. UPDRS-III score was 19 (see Table 1, video 1) and Hoehn and Yahr scale was stage 2. There was no evidence of decline in speech or cognitive function; the patient had no complaints and was more independent than previously for administrative tasks. Of note, the urinary incontinence initially improved but recurred at one year along with urinary urgency. Orthostatic hypotension persisted but was well controlled with 20 µg of fludrocortisone daily, and hallucinations did not recur.

## Discussion

To our knowledge, four other cases of STN DBS with SNCA mutations have been reported (three with SNCA duplication, one with a c.158c.A (p.A53E) missense mutation) along with one case of GPi stimulation [5,7,8,9,10].

In the first case of SNCA duplication, the female patient developed PD at 41 years of age and initially responded to dopaminergic treatment, but she developed motor fluctuations within two years as well as impulse control disorders. Bilateral STN DBS was implanted at the age of 46, with good motor outcome. Postoperative neuropsychological assessment showed decline in verbal fluency and shifting attention tests, as well as improvement in memory, attention and abstract reasoning. Depression scores improved. She died of unrelated cause 1.5 years after surgery, with her PD still controlled with 400 mg per day of LD split in 3 doses [7].

In the second case of SNCA duplication, the male patient had a positive family history of parkinsonism and psychiatric issues and presented symptoms of PD at the age of 35. He responded well to dopaminergic medication initially and developed motor fluctuations two years later. He had no obvious dementia nor depression and was implanted with bilateral STN DBS at the age of 41. This improved motor fluctuations and levodopa doses were reduced to 600 mg in three doses ten years after surgery. There was no evidence of dementia or psychiatric issues at follow-up. Motor outcomes were good after 10 years of follow-up [8].

In the third case of SNCA duplication, the patient developed symptoms at the age of 37 and was implanted with bilateral STN DBS at the age of 43. She had no family history of PD. Motor outcomes were good after 6.5 years of follow-up on 370 mg of LD in two doses per day and there was no cognitive decline [5].

In the case of the missense mutation, the female patient developed PD at 42 and levodopa was initiated two years later with a good response. The patient presented with motor fluctuations one year later and was implanted with bilateral STN DBS at the age of 47, which improved motor fluctuations and reduced required levodopa doses. This benefit persisted during the 3.5 years of follow-up. Neuropsychological testing at the age of 45 was normal. However, she developed cognitive and psychiatric symptoms in the years following the surgery, as well as deterioration of axial motor symptoms, and the global outcome was less favorable compared to the SNCA duplication cases described above [5,9].

One additional case of DBS is reported in a male patient with SNCA duplication, this time in the globus pallidus internus (GPi). The patient had no family history and presented initial PD symptoms at the age of 18. He initially responded to dopaminergic medication but soon developed motor fluctuations as well as mild cognitive decline and behavioral disorders and was implanted at 26. The GPi was selected as a target due to cognitive decline and to disabling dyskinesias. One month after the surgery, motor symptoms improved and peak-dose dyskinesias had resolved [10].

In our patient’s case, the decision for DBS was based on disabling motor complications and severe orthostatic hypotension. Bilateral STN was selected as a target in the absence of cognitive decline. The GPi was not the best target in our patient, as it would not have allowed us to reduce levodopa doses and therefore orthostatic hypotension would have persisted. In this specific case, orthostatic hypotension was levodopa-induced, and was managed easily after surgery. Additionally, dyskinesia did not recur under STN DBS, which was a risk, although it was not described in the above-mentioned cases.

This case illustrates that in SNCA duplication, presence of early motor complications and of levodopa-induced hypotension is not a contraindication. Provided the patient has no cognitive decline, DBS can improve quality of life. To our knowledge, our case is the only one in which the patient received no anti-Parkinson’s medication one year after surgery, at which time the outcome was still beneficial without evidence for cognitive or psychiatric decline. However, this is a one-year and therefore short follow-up. Long-term data and larger sample sizes are needed to draw further conclusions, but subthalamic nucleus deep brain stimulation may be a safe treatment for patients with SNCA mutation who experience disabling motor symptoms and severe LD-induced hypotension.
